# Research trends and hotspots evolution of cardiac amyloidosis: a bibliometric analysis from 2000 to 2022

**DOI:** 10.1186/s40001-023-01026-5

**Published:** 2023-02-20

**Authors:** Zhenyue Fu, Jiayu Lv, Xiya Gao, Bingxuan Zhang, Yumeng Li, Xia Xu, Haoran Zheng, Huaqin Wu, Qingqiao Song

**Affiliations:** 1 Department of General Internal Medicine, Guang’anmen Hospital, China Academy of Chinese Medical Sciences, Beijing, China; 2 Department of Cardiology, Guang’anmen Hospital, China Academy of Chinese Medical Sciences, Beijing, China; 3 Present Address: Beijing University of Chinese Medicine, Beijing, China

**Keywords:** Cardiac amyloidosis, AL amyloidosis, Transthyretin amyloidosis, Bibliometric analysis, Heart failure

## Abstract

In the new century, cardiac amyloidosis has received more attention from many countries and institutions, leading to innovations in the essence of the pathology, biological markers, noninvasive tests, and staging diagnoses and treatments for this disease. However, few reviews have summarized the research trends and hotspots in cardiac amyloidosis. Bibliometrics analysis is a statistically based approach to research that visualizes the contributions of academic institutions and changes in research hotspots. Therefore, in this paper, we used Citespace and VOSviewer software to conduct co-occurrence analysis and collaborative network analysis on the countries, institutions, and authors in the articles related to cardiac amyloidosis since the new century. And further find out burst keywords and references to obtain the research history, disciplinary development, and new hotspots and topics.

**Supplementary Information:**

The online version contains supplementary material available at 10.1186/s40001-023-01026-5.

## Introduction

When faced with the topic of heart failure, abnormalities in the structure and function of the heart are the first to be considered. However, cardiac amyloidosis (CA), an infiltrative myocardial interstitial change, has also shown a close association with heart failure [1]. The accumulation of abnormal β-folded proteins deposited in the myocardial interstitial forms amyloid fibrils that alter the normal structure of the myocardium, which in turn affects cardiac function and exhibits symptoms associated with restrictive cardiomyopathy and heart failure [2, 3]. However, the pathological essence of abnormally folded proteins is varied and proteomics has shown that more than 30 proteins can now form amyloid deposits [4]. The most frequent sources are immunoglobulin light chains (AL) produced by disordered plasma cells in the bone marrow, mutant transthyretin (ATTR-mu), or wild-type transthyretin (ATTR-wt) formed by dissociation of hepatic TTR [5]. The characteristic apple-green birefringence of myocardial tissue on Congo red staining is a uniform trait of different pathological essences of amyloidosis [6]. However, the variability of its pathophysiological substrate, the non-specific nature of its clinical manifestations (heart failure, arrhythmias, syncope, left ventricular hypertrophy), and the multi-organ involvement (kidney, nerve, soft tissue) leads to a low clinical detection rate, difficult differential diagnosis, and variable treatments for CA [7]. Meanwhile, as a rare disease, CA has a high mortality and disability rate and high medical costs. Median survival for AL combined with heart failure was only 6 months [8] and the median survival for ATTR was 26–67 months, mostly ending in sudden cardiac death and refractory heart failure [9]. A real-world study showed that the incidence of AL amyloidosis in the United States is increasing yearly, with 14.0 cases per million person-years [10], a mortality rate of approximately 19.7% in 1 year [11], and healthcare costs of approximately $114,030 [12]. By 2018, an estimated 74,000 cases of AL amyloidosis were diagnosed worldwide [13].

Since the twenty-first century, the field of CA has been studied in various ways, with a wide range of research directions and a large volume of articles output, and many high-quality studies and high-impact results have emerged. Therefore, it is necessary to review the research history and disciplinary development of CA from a scientific, professional, and objective perspective and seek new hotspots and topics based on the existing extensive literature.

Bibliometric analysis is a research method that uses statistical methods to quantitatively analyze various aspects of publications. The research results based on bibliometric analysis can be visualized in the form of figures and tables to obtain the development history, research progress, and emerging topics of a discipline, and highlight the contributions of various research teams/institutions/countries. Through bibliometric analysis, we can obtain development and advancement, fill the academic gaps, and break the bottleneck. The research on CA can be dated back to 1939, and after more than 80 years of research, we have gained a deeper understanding of this disease. To clarify the development history and research status, we will use Citespace and Vosviewer software to conduct bibliometric analysis and present the research trends and hotspots in a comprehensive, scientific, and intuitive way with figures and tables, which may provide evidence for guideline construction and future academic trends.

## Methods and materials

### Data sources and search strategy

**Fig. 1:**
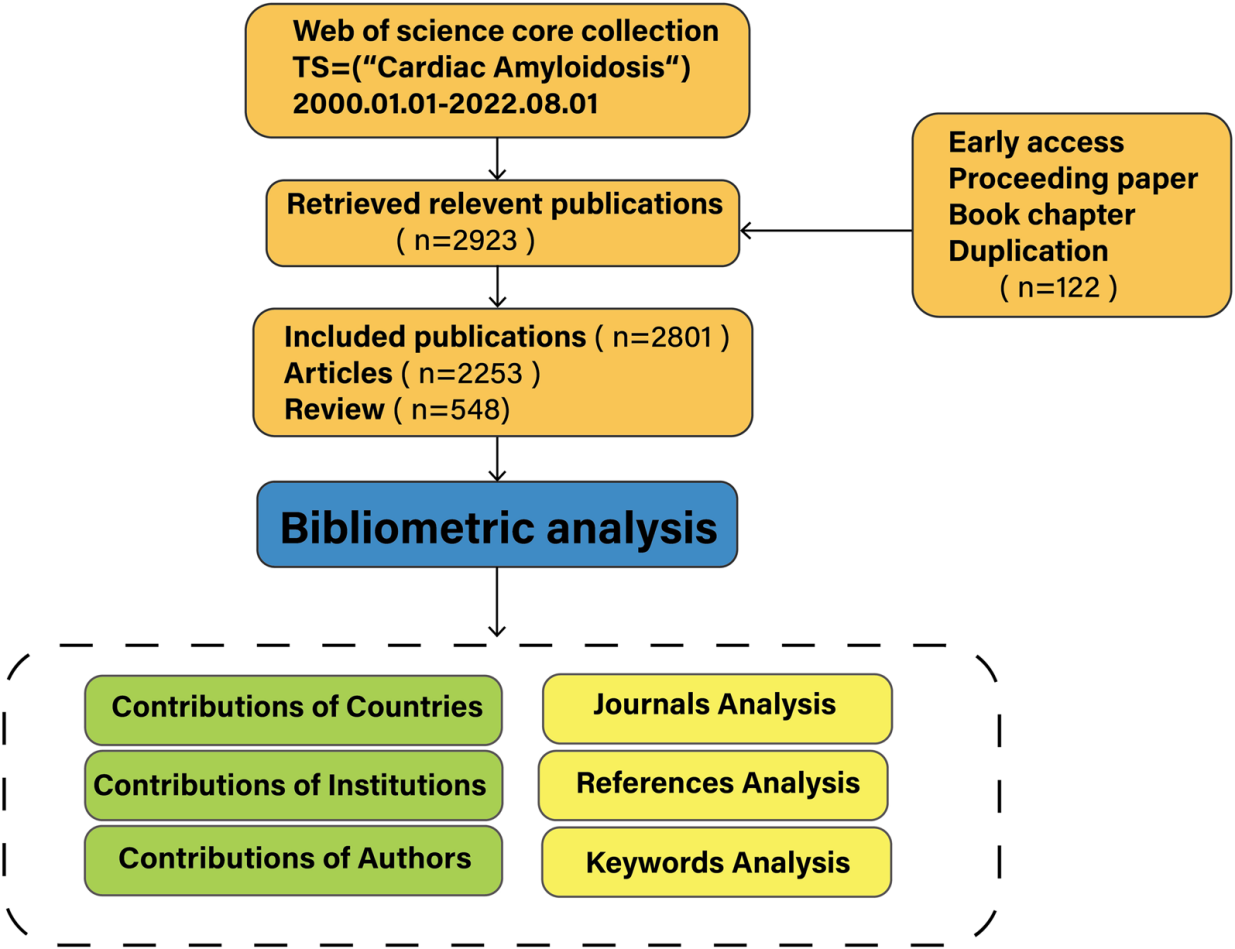
Flow chart of bibliometric analysis based on cardiac amyloidosis

Web of science is a large global, comprehensive, multidisciplinary, and high-impact academic information repository, which covers articles in many fields such as natural sciences, biomedicine, engineering, and technology. In this study, the science citation index expanded (SCI) database in the field of biomedicine was selected for the search to obtain the most comprehensive and accurate publications on CA. Information retrieval was conducted by FZY and GXY in the Web of Science Core Collection. The search edition range was set to the science citation index expanded (SCI) and the search terms were set to TS = (“cardiac amyloidosis”), the publication types were articles and review articles, the language of publication was English, and the search time range was from 2000.01.01 to 2022.08.01. The final number of search results was 2923 publications (Fig. 1).

### Literature screening and data cleaning

The 2923 publications downloaded from the Web of Science were censored, excluding (1) early access; (2) proceeding paper; (3) book chapters and other non-articles/reviews articles; (4) duplicate articles, to obtain the final articles included in the bibliometric analysis. Extract relevant information (the number of publications and co-citations, H-index, year of publication, country/region, institution, author, journal, keywords, and references), merge synonyms, and then incorporate the data into Citespace and VOSviewer software.

### Data analysis

In this bibliometric analysis, we mainly used Citespace (5.8R5) and VOSviewer (1.6.16) to visualize and analyze the data. In addition, “bibliometrix” and “gg2plot” packages of R software were also used for data analysis and figure plotting, and Scimago Graphica was used for the global geographic visualization of the publications.

## Results

### Annual publications

**Fig. 2:**
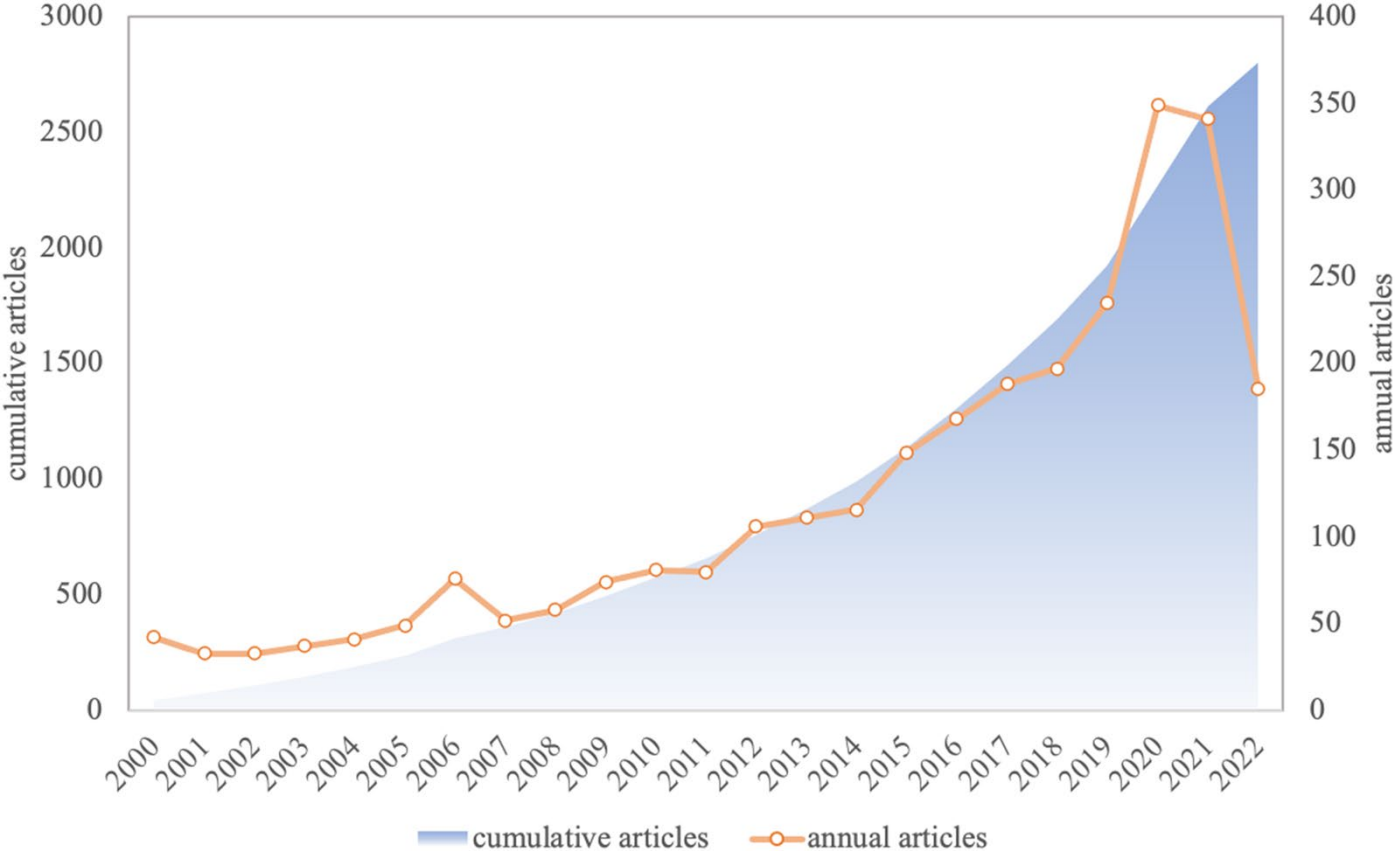
The trend of the annual published articles and cumulative articles of cardiac amyloidosis from 2000 to 2022

In this bibliometric analysis on CA during 2000–2022, we retrieved a total of 2801 publications, including 2253 articles and 548 reviews, accounting for 80.44% and 19.56%. Since 2000, the number of publications on the topic of "cardiac amyloidosis" has been mainly on the rise. There are three main phases: (1) stable growth period: between 2000 and 2011, the annual volume of publications fluctuated slowly, with a small increase in 2006, but the annual volume of publications was below 90; (2) rapid increase period: between 2012 and 2022, the annual volume of publications increased significantly, exceeding 100 publications for the first time in 2012 and reaching a peak of 349 publications in 2020; (3) plateau period: in 2021, there is a small decrease in the number of publications issued compared with the previous year. 2022 is only included until August, but the number of publications also did not see a significant increase. After decades of exploration, experiments, and argumentation, the research related to CA has come to a relatively mature stage (Fig. 2).

### Contribution of Countries/Institutions

**Fig. 3:**
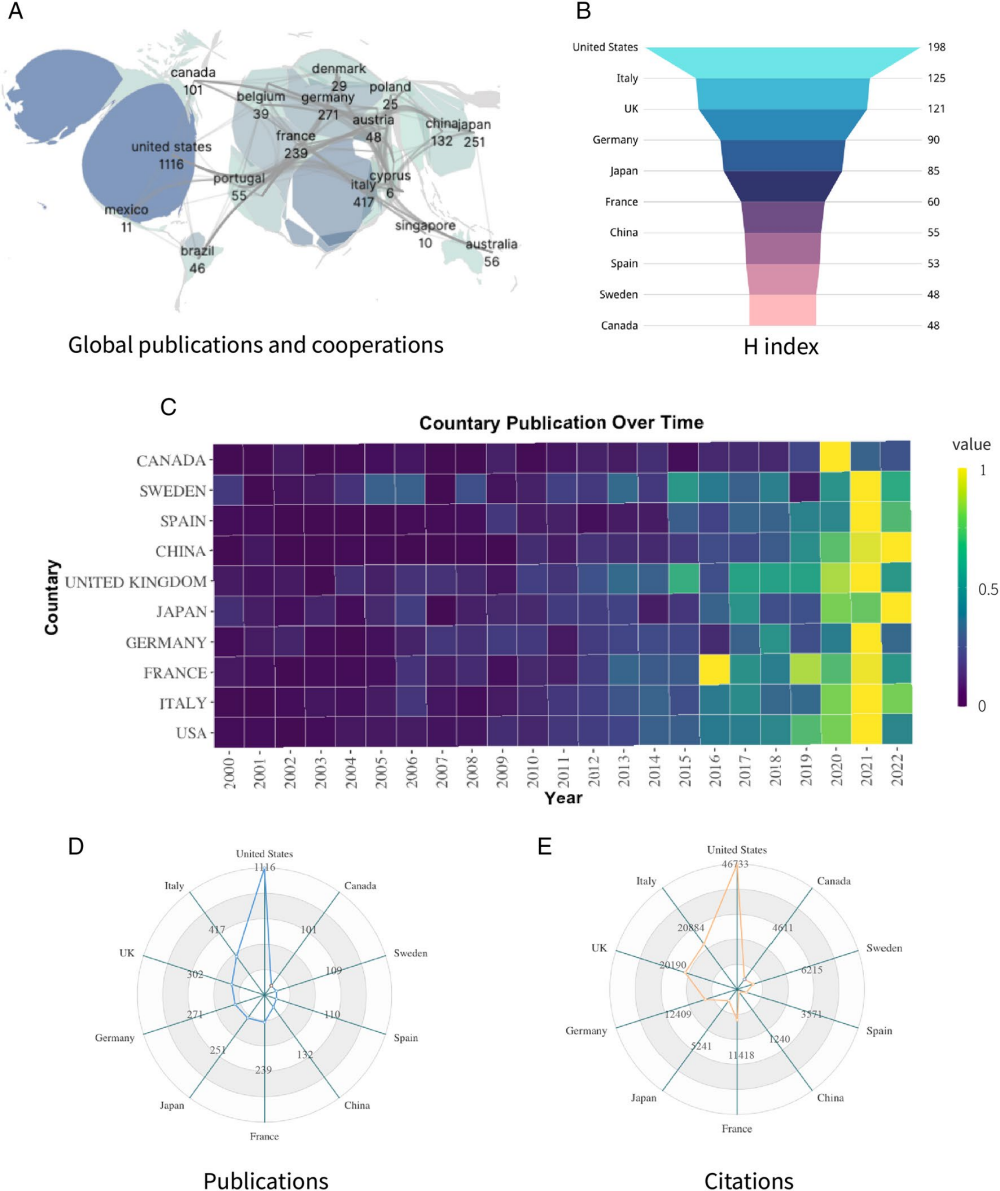
**A**. National publications; **B**. Intensity of collaboration; **C**. Thermal map of annual national publication volume; **D**. Number of publications; E. Number of citations

A total of 69 countries have published articles related to CA since the new century. The number of countries with cumulative publications greater than 5 publications is 44. The USA has the highest cumulative number of publications (1116/30%), citations (46733), and H-index (198), followed by Italy (417/11.2%) and the UK (302/8.1%) (Fig. 3B, D, E). Other European countries (Germany and France) and Asian countries (China and Japan) are also showing increasing interest in research. (Fig. 3 A, C).

**Fig. 4:**
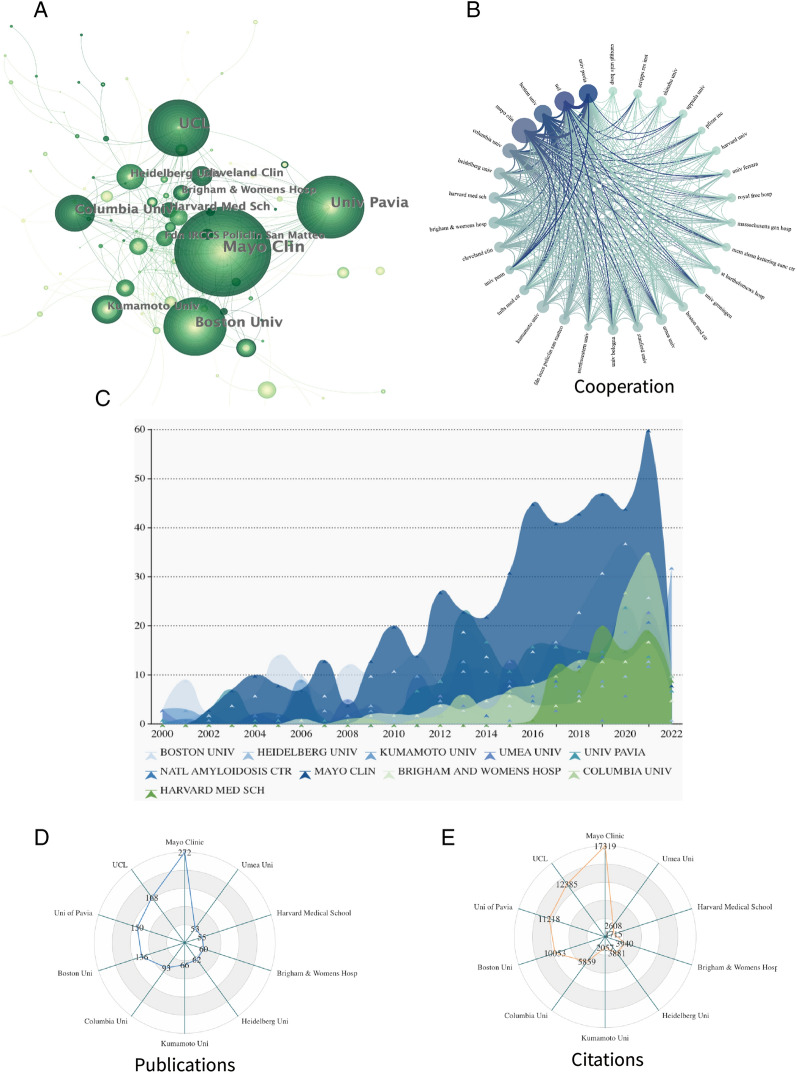
**A**. Occurrence of institutions; **B**. Intensity of collaboration; **C**. Map of annual publication volume; **D**. Number of publications; **E**. Number of citations

A total of 2836 institutions are involved in CA research. Figure 4B shows the top 30 productive institutions, among which Mayo Clinic is the first in the stratum with 272 cumulative publications and 17,319 citations (Fig. 4A, D, E). The Mayo Clinic is the center of academic collaboration, forming a global collaborative network of institutions with high-intensity cooperation, close academic ties, and frequent academic interactions. Figure 4C shows the changes in the volume of publications issued by the top ten institutions, and it can be found that the volume of publications issued by each institution has gradually increased.

### Contribution of authors

**Fig. 5:**
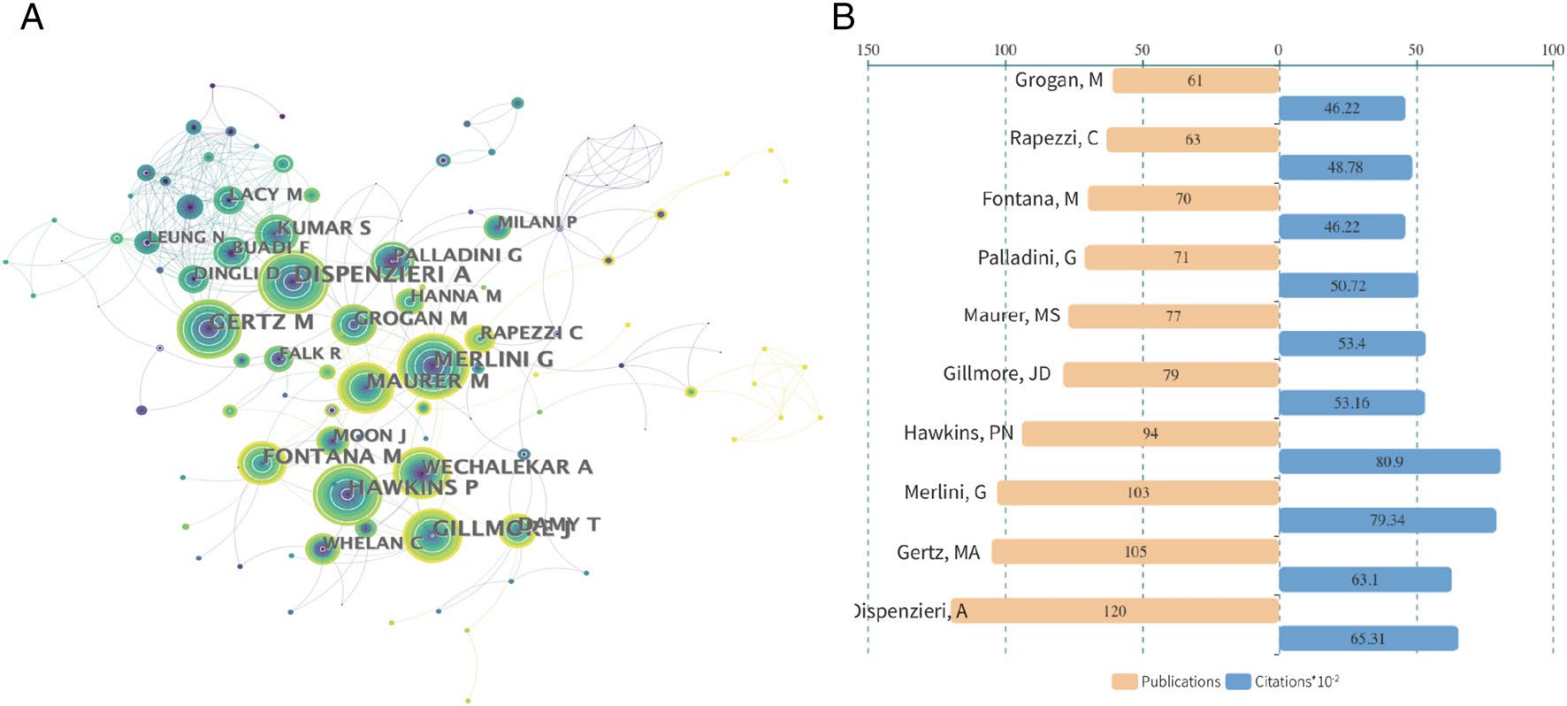
**A**. Occurrence of authors; **B**. Publications and citations of authors

A total of 12,753 authors are involved in the writing of publications related to CA. The top 10 authors are all high impact with H-indexes over 35, and Dispenzieri, A, Gertz, MA, and Merlini, G are the top three authors with 120, 105, and 103 publications, respectively.(Table 1) It is worth mentioning that Hawkins, PN has the highest H-index of over 60 and an uppermost average citation of 86.06 (Fig. 5A, B).

**Table 1:** Ranking of the top 10 productive authors in the field of cardiac amyloidosis

	Author	TP	TC	Avg.C	H-index	Country
1	Dispenzieri, A	120	6531	54.425	51	USA
2	Gertz, MA	105	6310	60.0952	52	USA
3	Merlini, G	103	7934	77.0291	52	ITALY
4	Hawkins, PN	94	8090	86.0638	60	UK
5	Gillmore, JD	79	5316	67.2911	45	UK
6	Maurer, MS	77	5340	69.3506	38	USA
7	Palladini, G	71	5072	71.4366	39	ITALY
8	Fontana, M	70	4622	66.0286	38	UK
9	Rapezzi, C	63	4878	77.4286	35	ITALY
10	Grogan, M	61	4622	75.7705	36	USA

*TP* total publications, *TC* total citations, *Avg.C* average citations

### Journals analysis

A total of 638 journals published publications related to CA, of which 132 journals had more than 5 publications. From the top 10 productive journals, we can find that the *Amyloid-journal of protein folding disorders* topped the list with a total output of 189 publications and a total citation of 3738. Followed by *the Journal of Nuclear Cardiology* and *Blood*, with 59 and 55 relevant publications. Among the top 10 journals, *Circulation* has the highest impact factor of 39.918, which confirms the excellent level of the top 10 journals (Table 2). However, in terms of the H-index, *Blood* (44) and *Amyloid-journal of protein folding disorders* (34) are two journals that have had a profound impact on the development of the discipline. *ESC Heart Failure* has gained momentum in the last 2 years in terms of average annual volume, while *Circulation* and *Journal of clinical oncology* top the list in terms of average citations.

**Table 2:** Ranking of the top 10 productive journals in the field of cardiac amyloidosis

	Journal	TP	TC	Avg.C	H index	IF
1	Amyloid-Journal of Protein Folding Disorders	189	3738	19.7778	34	6.571
2	Journal of Nuclear Cardiology	59	1014	17.1864	18	3.872
3	Blood	55	5826	105.9273	44	25.476
4	American Journal of Cardiology	46	1608	34.9565	23	3.133
5	ESC Heart Failure	46	307	6.6739	9	3.612
6	JACC-Cardiovascular Imaging	43	3361	78.1628	29	16.051
7	Journal of the American College of Cardiology	36	4162	115.6111	31	27.203
8	Circulation	31	6237	201.1935	25	39.918
9	European Heart Journal	30	3017	100.5667	24	35.855
10	American Journal of Hematology	27	1733	64.1852	17	13.265

*TP* total publications, *TC* total citations, *Avg.C* average citation

**Fig. 6:**
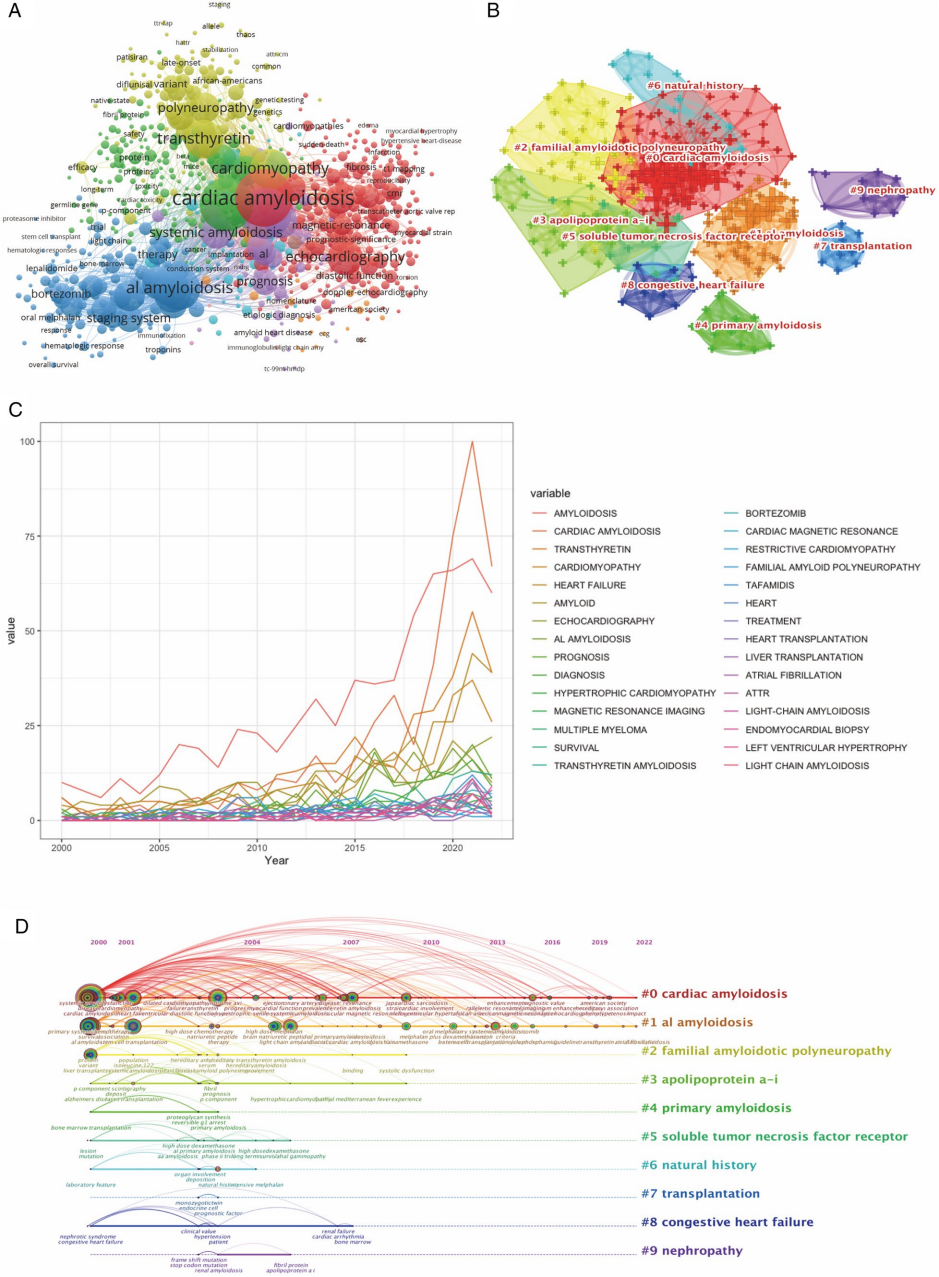
**A**. Keywords co-occurrence; **B**. Keyword clustering; **C**. Keyword annual evolution trend; **D**. Timeline of keywords

### Keywords analysis

A total of 6021 keywords were extracted from the publications, among which 416 appeared more than 10 times (Fig. 6A). It was observed that cardiac amyloidosis as the name of the disease was the core of all keywords, with the highest frequency (894 occurrences) and the most extensive association with other keywords (TLS6380). As shown in Fig. 6C, Al amyloidosis, transthyretin, and echocardiography associated with disease typology, pathological essence, and diagnosis were frequently mentioned. Clustering analysis of keywords allowed us to observe that 10 cross-over clusters, mostly related to disease name and classification (#0 CA, #1 al amyloidosis, #4 primary amyloidosis), pathological essence (#3 apolipoprotein a-i), the molecular mechanism (#5 soluble tumor necrosis factor receptor), treatment (#7 transplantation (liver/heart)), disease manifestations (#2 familial amyloidotic polyneuropathy, #8congestive heart failure, #9 nephropathy), and other correlations (Fig. 6B). In the timeline figure, it can be observed that the core terms of each cluster have different levels of interest in each period, with some topics persisting and developing more research directions over time (#0 Cardiac Amyloidosis, #1 al amyloidosis) and others fading (#5 soluble tumor necrosis factor receptor, #9 nephropathy) (Fig. 6D).

**Fig. 7:**
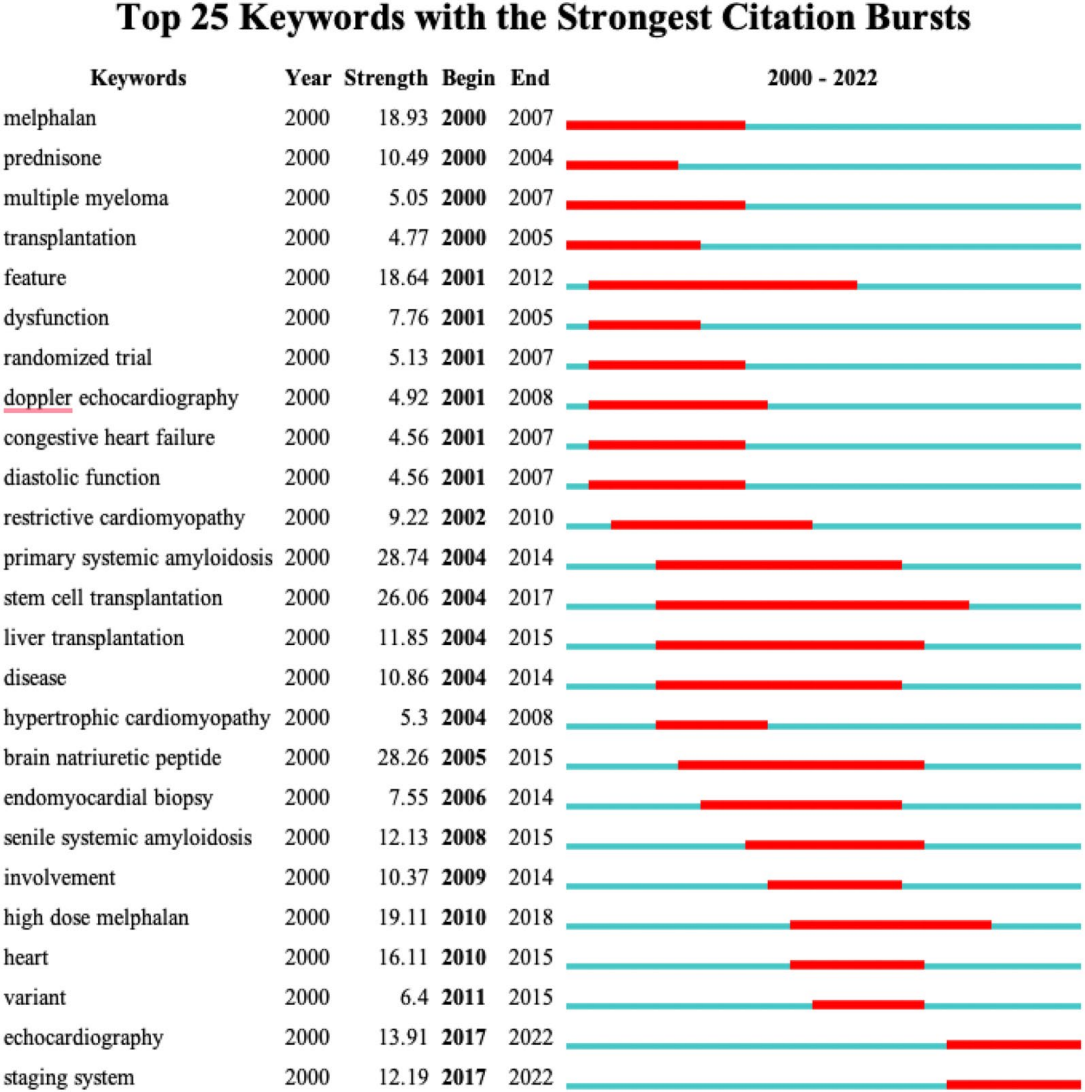
Top 25 keywords with the strongest citation bursts

The top 25 keywords are burst by Citespace software to sort out the hotspots and research trends related to CA since the new century (Fig. 7). From the timeline figure of CA burst keywords, we can find most burst keywords occurred in the stable growth period (2000–2011), mostly related to the topics of diagnosis and treatment such as “doppler echocardiography”, “melphalan”, “transplantation”, and so on. Although the output at this stage was relatively small, the large number of burst keywords provided the direction and benchmark for the subsequent in-depth study of CA and the arrival of the rapid rise period. It is worth mentioning that the keywords “stem cell transplantation” and “liver transplantation” have existed for a long period, which indicates that transplantation therapy has been the focus of researchers for a long time.

### Reference analysis

**Fig. 8:**
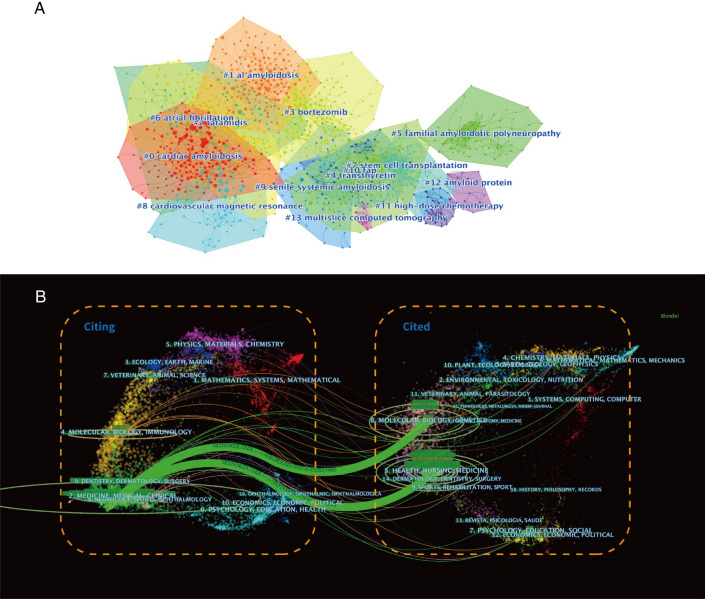
**A**. Reference clustering; **B**. A dual-map overlay of the journals on cardiac amyloidosis research

From the journal overlay map, we can observe that the citing literature is mainly concentrated in the *medicine, medical, and clinical* subject categories, and the cited literature is mainly concentrated in the *molecule, biology, genetics* and *health, nursing, and medicine subject* categories. This suggests that the research is focused on the pathological mechanisms of disease, medical therapy, and clinical research (Fig. 8B).

A total of 41,173 references were extracted, of which 860 references were cited more than 20 times. Clustering analysis of references to explore the research direction reveals that most of the references revolve around pathological essence (“transthyretin” and “amyloid protein”), disease typing (“al amyloidosis”, “familial amyloidotic polyneuropathy”, and “senile systemic amyloidosis”), treatment (“bortezomib”, “tafamidis stem cell transplantation”, and “high-dose chemotherapy”) (Fig. 8A). Analysis of the top 25 cited references showed that most of them were published in high-impact journals such as *The New England Journal of Medicine* (5), *Circulation* (5), and *Blood* (3). (Additional file 1: Table S1).

**Fig. 9:**
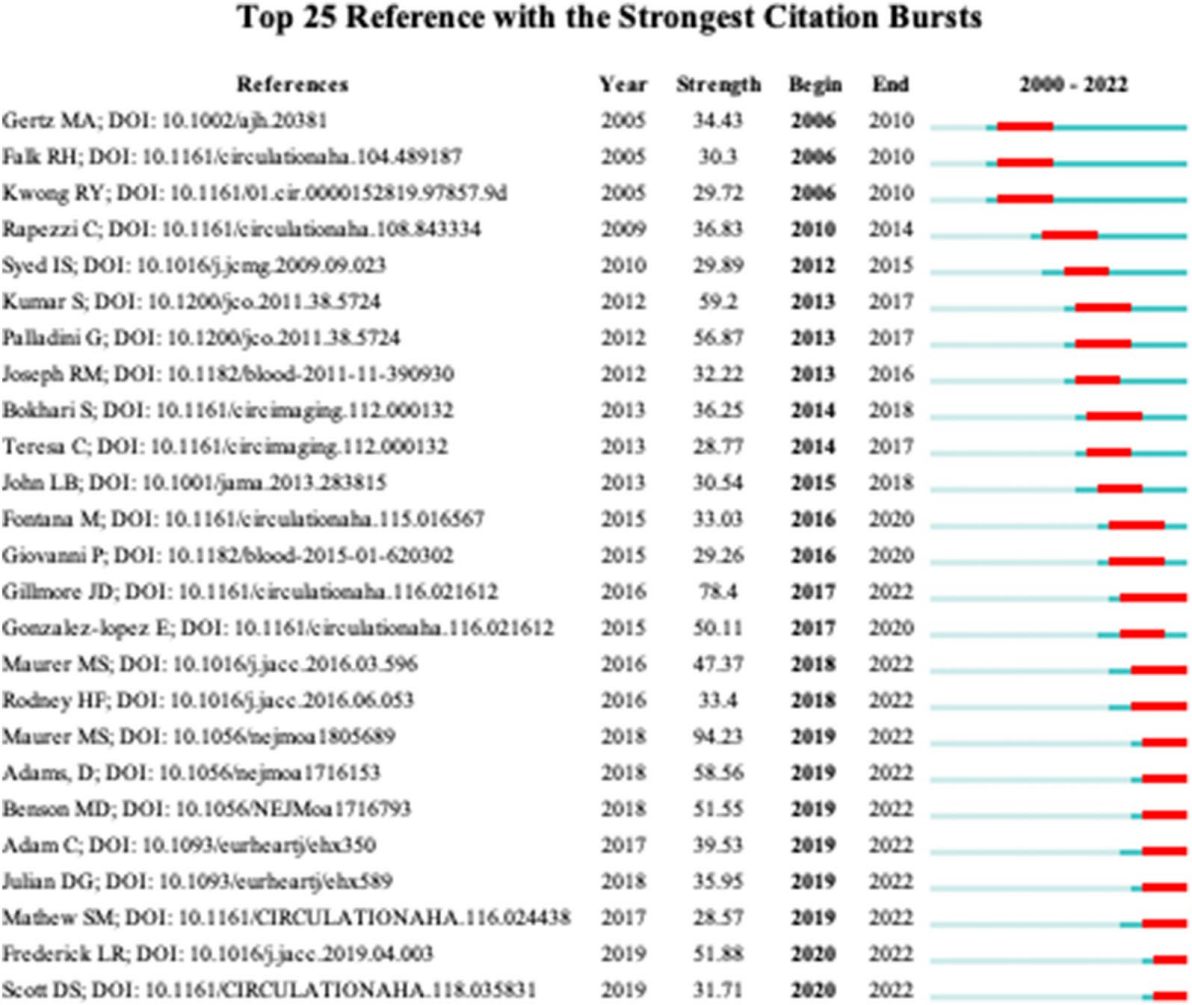
Top 25 references with the strongest citation bursts

At the same time, the burst analysis of the references showed that the top 25 references obtained from the burst analysis overlapped with 9 of the top 25 cited citations, and most of the duplicate citations were diagnostic, therapeutic, and review studies (Fig. 9). One of the consensuses published in the *American Journal of Hematology* harvested 502 citations. The paper by Gillmore JD et al. was ranked second with 442 citations and the paper by Dispenzieri A et al. was ranked third with 373 citations.

## Discussion and prospects

### Basic information

In this study, a bibliometric analysis with bibliometric software (Citespace, VOSviewer) was used to visualize the presentation and hotspot dissection of cardiac amyloidosis since the new century, showing in figures and tables the research contributions and collaborations of countries, institutions, and authors, the burst keywords and references, and hotspots evolution. From the Science Citation Index Expanded (SCI) database, 2801 relevant papers were retrieved, including 2253 publications and 548 reviews. The research related to CA has experienced a steady growth period (2000–2011) and a rapid rise (2012–2020) in the previous period and may be facing an academic plateau period at present. Notably, this growth trend coincides with the three stages of pre-science, conventional science, and scientific crisis in Thomas Kuhn's theory of *The Structure of Scientific Revolutions*. However, the observation time for the third stage is relatively short, and whether the research on CA enters the disciplinary plateau period needs to be based on a longer-term observation.

The USA dominates the core of the field with a large number (1116 publications) and high quality (H-index 198) of academic outputs and has formed academic cooperation networks with many European countries (Italy, UK, Germany). However, there is a lack of transnational collaboration with other countries and regions, so the globalization trend of interdisciplinary and academic exchange needs to be strengthened. There is a need for countries within the core academic network to take an active lead in building academic collaboration networks worldwide to conduct studies such as large-scale epidemiological surveys and large-scale multicenter clinical trials. Accordingly, institutions (Mayo Clinic, University College London, University of Pavia, etc.) and authors (Dispenzieri, A, Gertz, MA, Merlini, G, etc.) with outstanding contributions to CA have emerged in the above countries.

High-impact journals are the carriers of the publications, and the contribution of outstanding articles has a positive effect on the improvement of journal impact. Among the top 10 journals, we can find 6 journals with IF > 10 and 7 journals with an H index > 20. *ESC heart failure* has been a strong publication in the last 2 years, showing a strong interest in CA-related research.

### Research categories and potential hotspots

The keywords of the publication are highly refined by the authors of their academic results, guiding the research direction, academic topic, publication framework, and core thesis. Keyword frequency statistics, co-occurrence analysis, and cluster analysis can indicate the research themes and hotspots of CA in the new century, which plays a pivotal role for researchers to explore the changes and emerging trends in the discipline. Highly cited publications are seminal publications in a research field and their academic value can represent the authority of a field. A bibliometric analysis of highly cited literature can capture the current important scientific output in the field and grasp cutting-edge scientific dynamics. We sorted keywords and references and performed clustering and burst analysis to obtain hotspots and outstanding results and found that the main research points in CA were disease typing, diagnosis, differential diagnosis, and treatment. The burst keywords and references represent landmark academic results and popular research directions in CA. Their contents were sorted and extracted to obtain the popular research categories in a specific period. From the top 25 burst keywords and references in Citespace software, we obtained the following 4 research categories:

### Cardiac damage with other system involvement

Studies related to cardiac damage processes such as diastolic function, restrictive cardiomyopathy, and congestive heart failure in amyloidosis have been maintained at a high research fever level [14–16]. Diastolic dysfunction can be observed in CA patients on echocardiography (significant decrease of Ev velocity in all walls of the heart, lateral side < or = − 12 cm/s, medial side < or = − 10 cm/s,) [17, 18] In contrast, restrictive cardiomyopathy and hypertrophic cardiomyopathy may be phenotypes of CA, associated with amyloid deposits that infiltrate the myocardial interstitium and “sclerosis” and hypertrophy of the heart [19, 20] CA may be an under-recognized cause of heart failure, especially in HFpEF [21]. A meta-analysis showed that 11% of patients with HFpEF had transthyretin amyloid deposition and were associated with higher age and worse cardiological parameters (NT-proBNP, limb conduction hypovoltage, ventricular wall hypertrophy) [22]. Heart function  rapidly decrease in a short period, which produce an irreversibly poor prognosis [23]. Primary systemic amyloidosis, an abnormal proliferation of plasma cells, is also popularly studied. The heart is one of the sites of involvement of primary systemic amyloidosis, as well as nerve (autonomic/peripheral nerve) [24], soft tissue (carpal tunnel syndrome, microphthalmia, nail dystrophy, rash) [25–28], digestive system (hepatomegaly, Splenomegaly) [29], kidney (nephrotic syndrome, proteinuria) [30], etc.

### Non-invasive examination

With the development of noninvasive tests (echocardiography, speckle tracking imaging, cardiovascular magnetic resonance, and 99mTc-PYP scintigraphy) [31–33], the diagnosis of CA is no longer entirely dependent on endomyocardial biopsy, and the clinical detection rate of CA has been improved. Echocardiography plays a pivotal role in the early diagnosis of CA. Biventricular enlargement, impaired atrial function with thrombus, thickening of cardiac structures (septum, ventricular wall), and reduced ejection fraction can be found in patients with CA [34]. Granular or scintillation-like changes in the ventricular muscle are characteristic of CA [35]. Speckle tracking imaging (STI) shows impaired global longitudinal strain and preserved apical longitudinal strain in ATTR-CA patients [36]. Cardiovascular magnetic resonance (CMR) with the diagnostic value of gadolinium hemodynamics in CA was first validated in 2005 [32], and a characteristic overall pattern of delayed subendocardial enhancement with gadolinium enhancement and subendocardial (42%), mid-wall (29%), and epicardial (18%) amyloid deposition can be seen in CA patients. The presence of transmural abnormality was associated with higher BNP and cTnI levels, higher LVMI/RVMI, more severe ventricular hypertrophy, and worse cardiac function class. Moreover, the transmural abnormality is an independent risk factor predicting death in patients with CA (HR = 5.4, *p* < 0.01) [37]. The sensitivity specificity of 99mTc pyrophosphate (99mTc-PYP) scintigraphy as a specific diagnostic method for ATTR was 97% and 100%, respectively [38].

### Clinical prediction models

Before 2012, clinical prediction models of CA were based on NT-proBNP and cTnI [39], which were mainly associated with the degree of cardiac involvement and had significant clinical significance. However, the pathological mechanisms of CA, especially AL, are also associated with abnormal immunoglobulin light chains produced by disordered plasma cells. As the study progressed, it was found that the degree of reduction of amyloidosis free light chain (FLC) was closely related to the improvement of survival [40]. The stratified risk with cTnT 0.025 ng/mL, NT-proBNP 1800 pg/mL, and FLC diff 18 mg/dL could well evaluate the median survival time and 5-year survival rate [41]. Some researchers used NT-proBNP 3000 ng/L and eGFR 45 ml/min as entry points to classify ATTR into three disease stages, and the model reflected adequate prognostic information by quantifying the degree of cardiac infiltration, RAAS, and output versus perfusion [42].

### Treatment

The treatment modalities for CA are closely related to the pathological nature of amyloid. The research on various drugs has gradually evolved from small-unit clinical trials to international, multicenter, double-blind, placebo-controlled trials, providing a higher level of evidence-based results. Currently, in addition to supportive care, AL therapy revolves around anti-plasmapheresis (steroids, high-dose melphalan, proteasome inhibitors, and immunomodulators), autologous stem cell transplantation; ATTR mainly using ATTR inhibitors (Patisiran, Inotersen), TTR stabilizers (Diflunisal, Tafamidis), liver transplantation, etc. Alternative treatments (heart transplantation and ventricular assist devices) are used in the end stage. Studies have shown that there are risks associated with ASCT, with a transplant-related mortality rate of 12–13%. Therefore, patients should be evaluated for eligibility before treatment and clarify the scope of indications, contraindications, and management of transplantation-related complications [43]. Among the chemotherapeutic agents for AL, CyBorD is widely studied. A 2012 study of CyBorD for 17 AL patients who had not yet received ASCT found that 71% of patients had a complete hematologic response, 24% had a partial response, and increased the indication for ASCT [44]. More extensive clinical data suggest an overall hematologic response rate of 60% for CyBorD, but a lower response rate for patients with end-stage cardiac involvement and those at high risk. [45] TTR stabilizers (Diflunisal, Tafamidis) stabilize thyroxine to prevent dissociation and amyloid fibril formation [46, 47]. Clinical evidence has shown that diflunisal increases neurological stability in patients with ATTR combined with neuropathy [48]. Tafamidis decreases all-cause mortality and cardiovascular-related hospitalizations, increases 6-min walk distance, and improves KCCQ-OS scores [49]. New therapeutic agents for ATTR are mainly centered around RNA interference therapy. In 2013, a new RNA interference (ALN-TTR01) Phase I clinical trial was conducted and observed a rapid, dose-dependent, and sustained reduction in transthyretin levels, providing preliminary evidence for achieving RNA interference therapy for mutant gene silencing [50]. Studies for Patisiran have progressed to Phase 3 clinical trials, with results showing that Patisiran reduces mean left ventricular wall thickness, overall longitudinal strain, and NT-proBNP, reducing the occurrence of the composite endpoints of cardiac hospitalization and all-cause mortality [51].

As well as 3 potential hotspots and challenges in the future:

### Disease typing and management

So far, researchers have gradually considered AL and ATTR as two different cardiovascular diseases because of their pathophysiological substrates, diagnostic methods, and differences in clinical manifestations [52, 53]. Therefore, forming a perfect staging diagnosis, treatment, and management will be a hot topic in the future. In particular, the development of appropriate clinical diagnostic and prognostic models in the era of big data will help clinicians to detect the disease and make medical decisions.

### Systemic amyloidosis

In the past 5 years, the research teams have surged in the study of amyloidosis, which has surpassed the research fever on CA. It suggests that some research teams are beginning to pay more attention to the fact that amyloidosis is a pathological change with multi-organ and multi-system involvement, and a comprehensive study can help identify commonalities, reduce tissue involvement, and manage the system.

### Development of specific targeted drugs

The current detection rate of CA has increased with the development of various screening tools, but the treatment of CA remains a major challenge and research hotspot. Currently, there are few drugs available for CA, and the clinical response rate and efficacy remain low. New drugs such as some monoclonal antibodies for AL (Daratumumab, CAEL-101 (11-1F4), etc.) are still in phase I clinical trials, and new drugs for ATTR (Eplontersen, Vutrisiran, etc.) are in phase III clinical trials and require more extensive clinical data to determine their efficacy [54–56]. Therefore, in the future, there is a need to strengthen the links between various national institutions to conduct international, multiplex, placebo, double-blind randomized controlled trials to verify the efficacy of new drugs.

## Conclusion

This bibliometric analysis examined the research history of cardiac amyloidosis since the new century with bibliometric software. A total of 2801 relevant papers were retrieved from Web of Science. The visualization software was used to analyze them and found that myocardial amyloidosis has developed through three periods and gradually matured. The United States was the core country with the highest number of publications, citations, and H index. It dominates the field and forms a network of academic collaborations with many European countries (Italy, the UK, and Germany). Many institutions (Mayo Clinic, University College London, University of Pavia) and individuals (Dispenzieri, A, Gertz, MA, Merlini, G) have also contributed significantly to the study of cardiac amyloidosis. We also found that most of the top ten productive journals have high impact factors, indicating the high academic value of cardiac amyloidosis research. Finally, we analyzed the keywords and references to obtain four research categories and three potential hotspots, to provide scientific ideas for researchers and clinicians.

## Supplementary Information

**Additional file 1**: **Table S1**. Top 25 cited references.

## Data Availability

All data generated or analyzed during this study are included in this published article and its additional information files.
