# Supplementary material for: Research trends and hotspots evolution of cardiac amyloidosis: a bibliometric analysis from 2000 to 2022
Source: Eur J Med Res. 2023 Feb 20;28:89. doi: 10.1186/s40001-023-01026-5 (PMC9940355; doi:10.1186/s40001-023-01026-5)
Supplement: Supplementary file 1 — Additional file 1: Table S1. Top 25 cited references. [file 40001_2023_1026_MOESM1_ESM.docx]

Additional File

# Additional Table

Additional Table S1 Top 25 cited references

| id | label | Journal | First author (year) | Citations |
| --- | --- | --- | --- | --- |
| 1 | Definition of Organ Involvement and Treatment Response in Immunoglobulin Light Chain Amyloidosis (AL): A Consensus Opinion From the 10th International Symposium on Amyloid and Amyloidosis | AM J HEMATOL | Gertz MA (2005) | 502 |
| 2 | Nonbiopsy Diagnosis of Cardiac Transthyretin Amyloidosis | CIRCULATION | Gillmore JD (2016) | 442 |
| 3 | Serum Cardiac Troponins and N-Terminal Pro-Brain Natriuretic Peptide: A Staging System for Primary Systemic Amyloidosis | J CLIN ONCOL | Dispenzieri A (2004) | 373 |
| 4 | Tafamidis Treatment for Patients with Transthyretin Amyloid Cardiomyopathy | NEJM | Maurer MS (2018) | 356 |
| 5 | Revised Prognostic Staging System for Light Chain Amyloidosis Incorporating Cardiac Biomarkers and SerumFree Light Chain Measurements | J CLIN ONCOL | Kumar S (2012) | 354 |
| 6 | Molecular Mechanisms of Amyloidosis | NEJM | Merlini G (2013) | 315 |
| 7 | New Criteria for Response to Treatment in Immunoglobulin Light Chain Amyloidosis Based on Free Light Chain Measurement and Cardiac Biomarkers: Impact on Survival Outcomes | J CLIN ONCOL | Palladini G (2012) | 307 |
| 8 | Systemic Cardiac Amyloidoses Disease Profiles and Clinical Courses of the 3 Main Types | CIRCULATION | Rapezzi C (2009) | 306 |
| 9 | Noninvasive Etiologic Diagnosis of Cardiac Amyloidosis Using 99mTc-3,3-Diphosphono1,2-Propanodicarboxylic Acid Scintigraphy | J AM COLL CARDIOL | Perugini E (2005) | 296 |
| 10 | Diagnosis and Management of the Cardiac Amyloidoses | CIRCULATION | Falk RH (2005) | 288 |
| 11 | Wild-type transthyretin amyloidosis as a cause of heart failure with preserved  ejection fraction | EUR HEART J | Gonzalez-lopez E (2015) | 263 |
| 12 | Cardiovascular Magnetic Resonance in Cardiac Amyloidosis | CIRCULATION | Maceira AM (2005) | 261 |
| 13 | Primary systemic amyloidosis: clinical and laboratory features in 474 cases | SEMIN HEMATOL | Kyle RA (1995) | 256 |
| 14 | Relative apical sparing of longitudinal strain using two-dimensional speckle-tracking echocardiography is both sensitive and specific for the diagnosis of cardiac amyloidosis | HEART | Phelan D (2012) | 235 |
| 15 | Genotype and Phenotype of Transthyretin Cardiac Amyloidosis | J AM COLL CARDIOL | Maurer MS (2016) | 233 |
| 16 | Patisiran, an RNAi Therapeutic, for Hereditary Transthyretin Amyloidosis | NEJM | Adams D (2018) | 224 |
| 17 | 99mTc-Pyrophosphate Scintigraphy for Differentiating LightChain Cardiac Amyloidosis From the Transthyretin-Related Familial and Senile Cardiac Amyloidoses | CIRC-CARDIOVASC  IMAG | Bokhari S (2013) | 219 |
| 18 | Serum N-Terminal Pro–Brain Natriuretic Peptide Is a Sensitive Marker of Myocardial Dysfunction in AL Amyloidosis | CIRCULATION | Palladini G (2003) | 218 |
| 19 | Inotersen Treatment for Patients with Hereditary Transthyretin Amyloidosis | NEJM | Benson MD (2018) | 202 |
| 20 | A European collaborative study of treatment outcomes in 346 patients with cardiac stage III AL amyloidosis | BLOOD | Wechalekar AD (2013) | 187 |
| 21 | Senile systemic amyloidosis affects 25% of the very aged and associates with genetic variation in alpha2-macroglobulin and tau: a population-based autopsy study. | ANN MED | Tanskanen M (2008) | 176 |
| 22 | Classification of amyloidosis by laser microdissection and mass spectrometry-based proteomic analysis in clinical biopsy specimens | BLOOD | Vrana JA (2009) | 172 |
| 23 | Repurposing Diflunisal for Familial Amyloid Polyneuropathy A Randomized  Clinical Trial | JAMA-J AM MED  ASSOC | Berk JL (2013) | 170 |
| 24 | High-Dose Melphalan versus Melphalan plus Dexamethasone for AL Amyloidosis | NEJM | Jaccard A (2007) | 164 |
| 25 | Incidence and natural history of primary systemic amyloidosis in Olmsted County, Minnesota, 1950 through 1989 | BLOOD | Kyle RA (1992) | 156 |
